# Non-collinear magnetism in the post-perovskite thiocyanate frameworks CsM(NCS)_3_†

**DOI:** 10.1039/d2sc06861c

**Published:** 2023-02-21

**Authors:** Madeleine Geers, Jie Yie Lee, Sanliang Ling, Oscar Fabelo, Laura Cañadillas-Delgado, Matthew J. Cliffe

**Affiliations:** a School of Chemistry, University Park Nottingham NG7 2RD UK matthew.cliffe@nottingham.ac.uk; b Institut Laue Langevin 71 avenue des Martyrs CS 20156 38042 Grenoble Cedex 9 France; c Advanced Materials Research Group, Faculty of Engineering, University of Nottingham, University Park Nottingham NG7 2RD UK

## Abstract

AMX_3_ compounds are structurally diverse, a notable example being the post-perovskite structure which adopts a two-dimensional framework with corner- and edge-sharing octahedra. Few molecular post-perovskites are known and of these, none have reported magnetic structures. Here we report the synthesis, structure and magnetic properties of molecular post-perovskites: CsNi(NCS)_3_, a thiocyanate framework, and two new isostructural analogues CsCo(NCS)_3_ and CsMn(NCS)_3_. Magnetisation measurements show that all three compounds undergo magnetic order. CsNi(NCS)_3_ (Curie temperature, *T*_C_ = 8.5(1) K) and CsCo(NCS)_3_ (*T*_C_ = 6.7(1) K) order as weak ferromagnets. On the other hand, CsMn(NCS)_3_ orders as an antiferromagnet (Néel temperature, *T*_N_ = 16.8(8) K). Neutron diffraction data of CsNi(NCS)_3_ and CsMn(NCS)_3_, show that both are non-collinear magnets. These results suggest molecular frameworks are fruitful ground for realising the spin textures required for the next generation of information technology.

## Abstract 2

The synthesis, structure and magnetic properties of the layered molecular post-perovskites CsM(NCS)_3_ M = Ni, Mn, Co, are reported. Neutron diffraction and bulk magnetometry show these isomorphic compounds order with different complex non-collinear magnetic structures.

## Graphical abstract

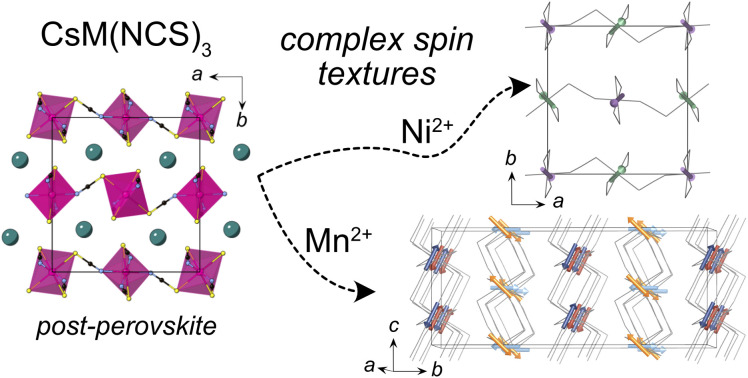

## 1 Introduction

A unifying goal in solid-state science is control over the physical properties of materials, and the tunability of perovskites is perhaps the most striking example. Traditionally these compounds are three-dimensional frameworks with a general chemical formula AMX_3_ built from [MX_6_] corner-sharing octahedra. Through substitution of the A, M and X ions, materials with remarkable magnetic,^1^ electronic conductivity,^2^ photovoltaic^3^ and non-linear optical properties^4^ have been created.

The perovskite structure is, however, only one of a wide-range of AMX_3_ structure-types, with perhaps the most closely related being the post-perovskite structure. In contrast to perovskites, post-perovskites contain [MX_6_] octahedra that both edge- and corner-share, resulting in two-dimensional anionic [MX_3_] layers, rather than a three dimensional framework. These layers are stacked with interstitial A cations positioned between them. Post-perovskites are amongst the most abundant terrestrial minerals, as the MgSiO_3_ perovskite that makes up much of the Earth's lower mantle undergoes a critical high-pressure and temperature phase transition (*P*_C_ ≈ 125 GPa, *T*_C_ ≈ 1250 K) to the post-perovskite structure-type near the mantle–core boundary.^5,6^ Due to the difficulty of reaching these extreme pressures, post-perovskites which form at more accessible pressures and can be recovered on quenching have proved useful analogues. These include the second- and third-row transition metal oxides AMO_3_, A = Na, Ca, and M = Pt, Rh, Ir (*P*_syn_ ≈ 5 GPa);^7–10^ and first-row fluorides NaMF_3_, M = Mg, Ni, Co, Fe, Zn.^11–15^ A handful of post-perovskite compounds can even be obtained at ambient conditions: notably CaIrO_3_,^16^ the post-actinide chalcogenides AMnSe_3_ (A = Th, U)^17^ and UFeS_3_,^18^ and TlPbI_3_.^19^ As a result of this synthetic challenge, systematic tuning of the properties of post-perovskites is much less well explored than for perovskites.

In particular, there are limits on our current understanding of the magnetic properties of post-perovskites, in part because neutron diffraction studies require large sample sizes. This is despite the fact that post-perovskites, unlike perovskites, tend to have non-collinear magnetic structures.^12,15,16,20^ As a result, both the exploration of fundamental properties of post-perovskites and the potential utility of their non-trivial spin textures for spintronic devices^21,22^ or quantum memory storage^23^ remains limited.

In contrast to the relative scarcity of atomic post-perovskites, molecular post-perovskites, where X is a molecular anion, are a growing class of materials.^24–28^ Unlike their atomic analogues, the majority of molecular post-perovskites are stable and synthesisable at ambient pressure. Incorporating molecular components in these frameworks can also allow for novel physical properties, arising from the additional degrees of freedom, including non-linear optics,^29^ electric polarisation^30,31^ and complex spin textures.^32^

Metal dicyanamides, AM(dca)_3_ dca = N(CN)_2_^−^, are the best established family of molecular post-perovskites.^33–35^ The metal ions are separated by five-atom-bridges and tend to be magnetically isolated, with no conclusive evidence of long-range magnetic ordering.^24,25,36^ To explore collective magnetic behaviour in molecular post-perovskite analogues we therefore focussed on ligands capable of propagating stronger superexchange interactions.

Thiocyanate (NCS^−^) is a promising molecular ligand for creating magnetic coordination frameworks with long-range magnetic ordering, even over extended distances.^37–42^ The thiocyanate also has asymmetric reactivity, unlike both atomic ligands and most common molecular ligands, *e.g.* formate or dca^−^.^43^ Applying the hard–soft acid–base principle,^44^ the nitrogen terminus is less polarisable and so coordinates preferentially to harder first-row transition metals, whereas sulfur has more diffuse orbitals, and preferentially coordinates to softer main group second- and third-row transition metals.^38^ Homoleptic framework structures are thus comparatively rare, beyond the binary M(NCS)_2_.^37,38^ Nevertheless, there are two reported AM(NCS)_3_ homoleptic frameworks with the post-perovskite structure: RbCd(NCS)_3_^45^ and CsNi(NCS)_3_.^26^

In this work, we study CsM(NCS)_3_, M = Ni, Mn and Co. We describe the synthesis of CsMn(NCS)_3_ and CsCo(NCS)_3_ and determine their structures, by single crystal X-ray diffraction, to be post-perovskites isomorphic with CsNi(NCS)_3_. We used bulk magnetometry to show that all three order magnetically. CsNi(NCS)_3_ (*T*_C_ = 8.5(1) K) and CsCo(NCS)_3_ (*T*_C_ = 6.7(1) K) order as weak ferromagnets with significant coercive fields, CsNi(NCS)_3_*H*_C_ = 0.331(2) T and CsCo(NCS)_3_*H*_C_ = 0.052(2) T, whereas CsMn(NCS)_3_ orders as an antiferromagnet *T*_N_ = 16.8(8) K. We then report neutron diffraction measurements, with single crystal and powder samples. These found CsNi(NCS)_3_ to be a canted ferromagnet, **k** = (0, 0, 0), and CsMn(NCS)_3_ to be a non-collinear antiferromagnet which orders with 
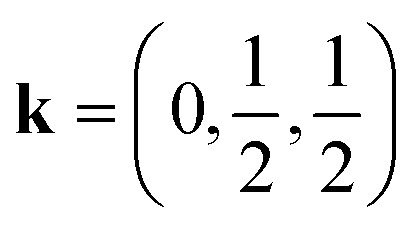
 and four sublattices which all order antiferromagnetically. Additionally, DFT calculations were undertaken (see ESI† for details), which confirm the intralayer interactions are at least an order of magnitude stronger than the interlayer interactions in these compounds.

Our results suggest that magnetic molecular frameworks, and thiocyanates in particular, are a promising ground for exploring the magnetism of otherwise challenging to realise structure-types. The non-collinear structures we have uncovered in these thiocyanate frameworks *via* neutron diffraction suggest that further studies of this family may uncover new routes to complex spin textures.

## 2 Results

### 2.1 Synthesis and structure

We synthesised CsNi(NCS)_3_, CsMn(NCS)_3_ and CsCo(NCS)_3_ by salt metathesis of the metal sulfate and caesium sulfate with barium thiocyanate in aqueous solution. CsNi(NCS)_3_ is stable at ambient conditions, however CsMn(NCS)_3_ and CsCo(NCS)_3_ are sensitive to humidity. Single crystals were obtained through slow evaporation and the structures were determined using single crystal X-ray diffraction to have the monoclinic space group *P*2_1_/*n*, and to crystallise in the post-perovskite structure. We found that, unlike the Ni and Mn analogues, the crystallisation of CsCo(NCS)_3_ from solution typically occurs in a two step process. Large, deep blue crystals of a second phase, believed to be Cs_2_Co(NCS)_4_, are typically obtained, which recrystallise into small, deep purple single crystals of CsCo(NCS)_3_ if left undisturbed for several weeks in ambient conditions.

**Fig. 1:**
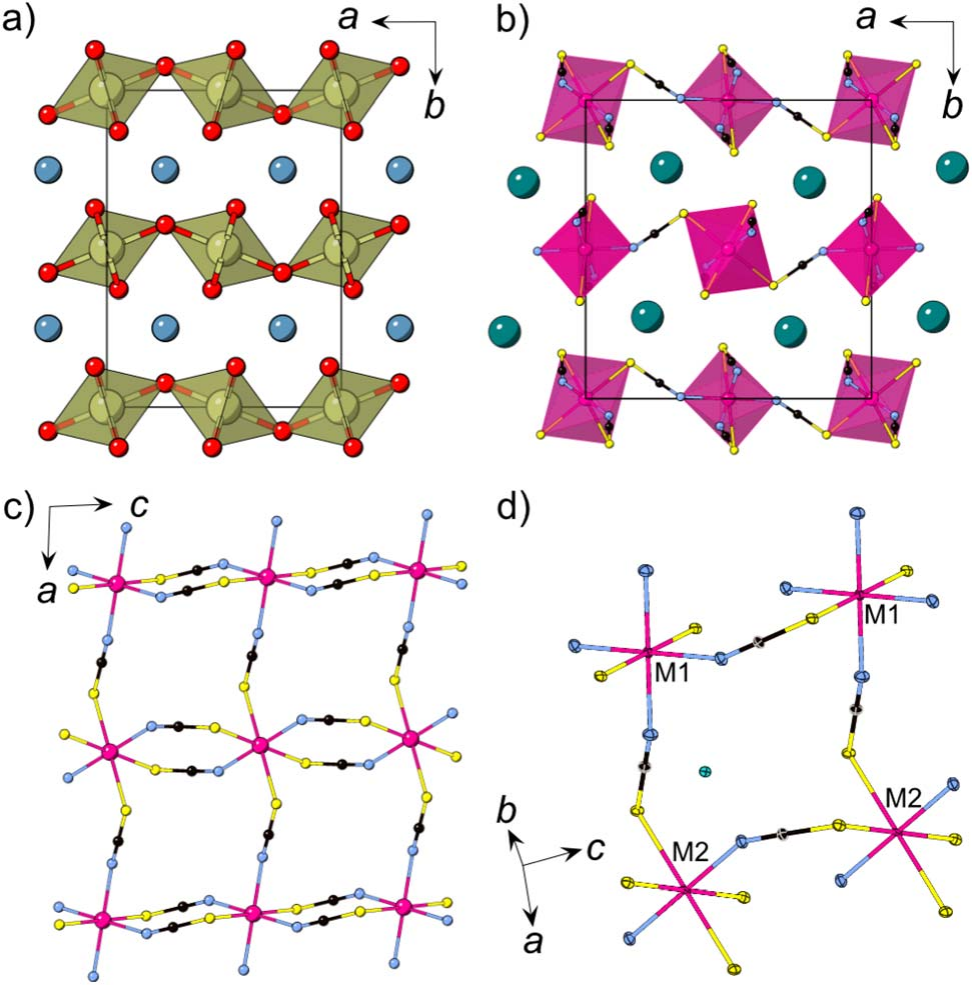
(a) The structure of CaIrO_3_ with iridium polyhedra connected *via* corner-sharing (*a* axis) and edge-sharing (*c* axis) [IrO_6_] octahedra, Ca = blue, Ir = brown, O = red. (b) Crystal structure of CsMn(NCS)_3_ at 120 K obtained from single crystal X-ray diffraction. (c) A single [Mn(NCS)_3_]^−^ layer. (d) Close-up view of the structure highlighting the two chemically independent metal ions (M1, M2). Cs = green, Mn = pink, N = blue, C = black, S = yellow.

All three compounds are isomorphic and consist of anionic [M(NCS)_3_] layers in the *ac* plane in which the transition metal M^2+^ ions are connected through *μ*_1,3_NCS ligands (Fig. 1c). In between the layers, which are stacked along the *b* axis, lie the charge balancing caesium counterions. The M^2+^ ions are octahedrally coordinated and there are two crystallographically and chemically distinct metal sites. One transition metal ion (M1, Wyckoff site 2*c*) is coordinated by four nitrogen atoms and two sulfur atoms, whilst the second transition metal ion (M2, Wyckoff site 2*b*) is bonded to four sulfur and two nitrogen atoms. The metal octahedra corner-share along the *a* axis and alternate between M1 and M2. Along the *c* axis, the metal octahedra edge-share, and all the metal sites within an edge-sharing chain are the same.

Moving along the row from manganese to nickel, the average M–N and M–S bond lengths, *d*_M–N_ and *d*_M–S_, decrease: *d*_Mn–N_(13 K) = 2.1607(14) Å, *d*_Co–N_(120 K) = 2.072(3) Å, *d*_Ni–N_(15 K) = 2.035(5) Å and *d*_Mn–S_ = 2.674(3) Å, *d*_Co–S_ = 2.5652(10) Å, *d*_Ni–S_ = 2.350(20) Å. The M–S bond lengths shorten to a greater extent than the M–N bond lengths, likely as a result of the higher polarisability of sulfur. This trend is comparable in other metal thiocyanate frameworks.^37,46^ There are two intralayer M⋯M distances, between the edge-sharing octahedra (*d*_M1–M1_ = *d*_M2–M2_ = *c*) and the corner-sharing octahedra (*d*_M1–M2_ = *a*). Again, moving from Mn^2+^ to Ni^2+^, the distances decrease, with *d*_Mn1–Mn1_ = 5.6540(4) Å compared to *d*_Co1–Co1_ = 5.57860(11) Å and *d*_Ni1–Ni1_ = 5.5409(6) Å; and *d*_Mn1–Mn2_ = 6.37315(5) Å, *d*_Co1–Co2_ = 6.30515(5) Å and *d*_Ni1–Ni2_ = 6.2631(6) Å. The interlayer spacing also decreases, with the shortest M⋯M distances, *d*_M,layer_, decreasing from *d*_Mn,layer_ = 7.20052(15) Å, through *d*_Co,layer_ = 7.18340(10) Å to *d*_Ni,layer_ = 7.1050(8) Å. For the single crystal neutron diffraction measurements of CsNi(NCS)_3_ a significantly smaller crystal was used in comparison to the CsMn(NCS)_3_, which influences the quality of the reflections, resulting in the larger errors.

In addition to this post-perovskite structure, there are two different perovskite structure-types with composition AM(NCS)_3_, CsCd(NCS)_3_ ^45^ and (NH_4_)_2_NiCd(NCS)_6_ (ESI Fig. 7†).^47^ To understand the relative stability of the post-perovskite structure compared to the perovskite-type structures, density functional theory (DFT) calculations were performed. Hypothetical perovskites were generated through atom-swaps, and the geometry optimised relaxation, relative to the experimentally observed post-perovskite structure, was calculated (Table 1). For simplicity, we focused on the spin-ferromagnetic solution of the three different structure-types for this comparison. We found that the post-perovskite structure was more stable than the perovskite structures by around 10 kJ mol^−1^ (0.1 eV per formula unit, approximately 4*kT* at room temperature). This energy is consistent with the observed exclusive formation of the post-perovskite structure, but suggests that more unusual experimental conditions may allow access to the perovskite phases.

**Table 1:** DFT predicted relative energies between the experimental post-perovskite phase (CsM(NCS)_3_ = pPv) and the two known AM(NCS)_3_ perovskite structure-types: CsCd(NCS)_3_ = Cs[Cd]; (NH_4_)_2_NiCd(NCS)_6_ = NH_4_[NiCd]. The perovskite structures were generated by swapping the A site cation for Cs^+^ and the B site cation(s) by the appropriate transition metal

M^2+^	*E*(pPv)^a^	*E*(Cs[Cd])^a^	*E*(NH_4_[NiCd])^a^
Ni	0.0	+90.8	+177.3
Mn	0.0	+84.8	+137.0
Co	0.0	+286.7	+114.5

a (meV per formula unit).

### 2.2 Magnetism

Having synthesised an isostructural series of thiocyanate post-perovskites containing paramagnetic ions, we next sought to understand their magnetic behaviour. We measured the bulk magnetic susceptibility of each of these compounds, in an applied field of 0.01 T, to determine the magnetic ordering temperature and average interaction strength. We used isothermal magnetisation measurements at 2 K over the range −7 to +7 T (−6 to +6 T for M = Ni) to assess the degree of magnetic hysteresis.

**Fig. 2:**
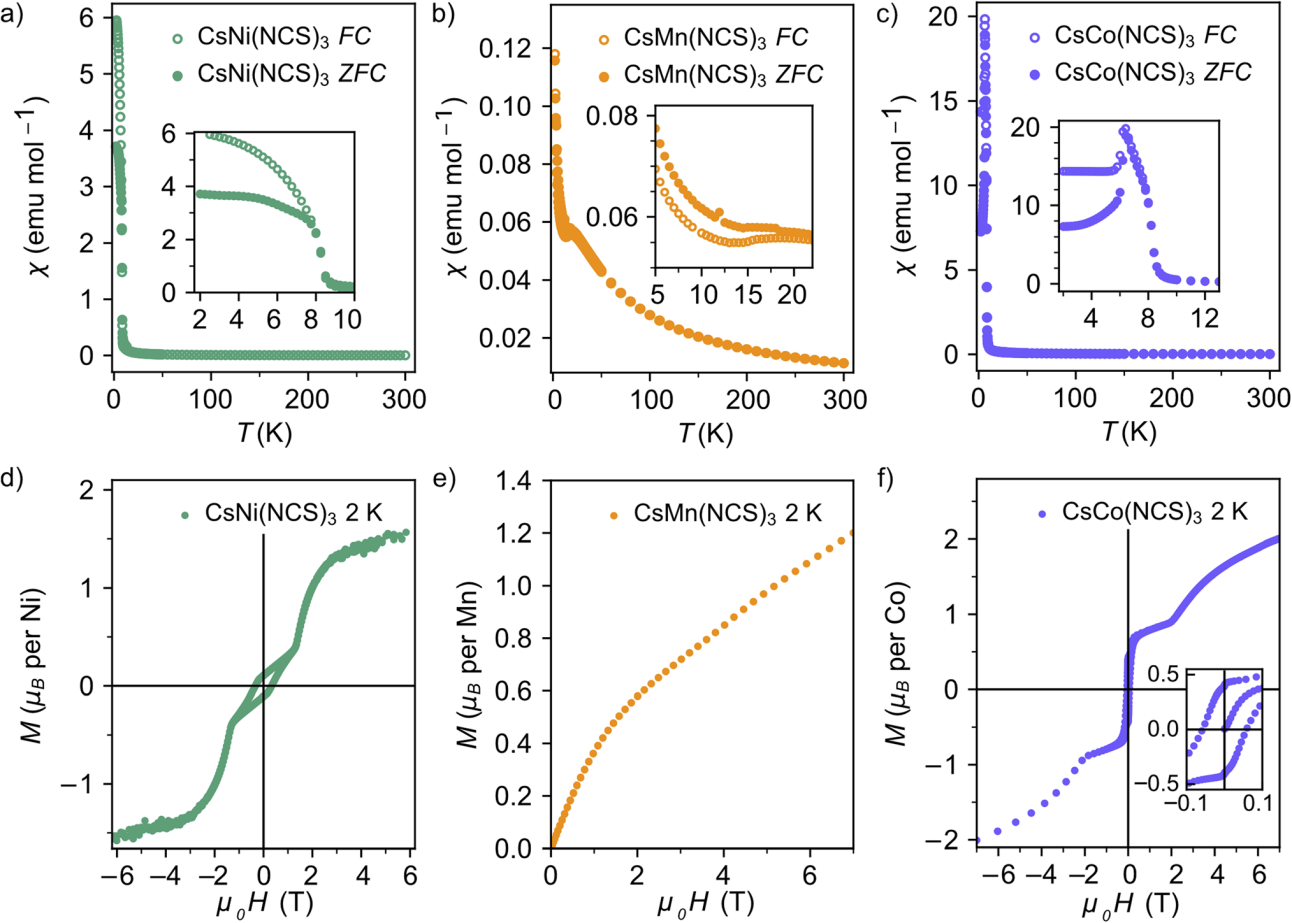
Magnetic susceptibility data with an applied field of 0.01 T for (a) CsNi(NCS)_3_; (b) CsMn(NCS)_3_; (c) CsCo(NCS)_3_; and isothermal magnetisation measurements at 2 K for (d) CsNi(NCS)_3_ (field −6 to +6 T); (e) CsMn(NCS)_3_ (field 0 to +7 T); and (f) CsCo(NCS)_3_ (field −7 to +7 T).

The zero-field cooled (ZFC) and field-cooled (FC) susceptibility of CsNi(NCS)_3_ diverge below the ordering temperature *T*_C_ = 8.5(1) K (Fig. 2a). The Curie–Weiss fit of the inverse susceptibility above 180 K gives a value for the Curie–Weiss temperature, *θ*_CW_, of −8.6(8) K. However, this value is particularly sensitive to the fitting temperature range: *θ*_CW_ = +1.5(4) K when 100 < *T* < 300 K, whereas *θ*_CW_ = −12.7(8) K for a fit 200 < *T* < 300 K. This variation is likely due to the presence of significant single-ion anisotropy, typical of Ni^2+^.^48,49^ The Curie constant, *C*, is 0.85(2) emu K mol^−1^, which is lower than the spin only value, *C*_spin only_ = 1 emu K mol^−1^. The lower than expected Curie constant (ESI Fig. 1†) is likely due to a sample mass error. The isothermal magnetisation of CsNi(NCS)_3_ at 2 K shows hysteresis with a coercive field of *H*_C_ = 0.331(2) T and a remnant magnetisation *M*_rem_ = 0.106(1) *μ*_B_ per Ni, implying a canting angle of 6.1° if there are only two distinct spin orientations. Beyond 1.19(1) T, the hysteresis loop closes and there is a magnetic phase transition to a second magnetic phase, reaching a magnetisation of 1.54(1) *μ*_B_ per Ni at 6 T.

The magnetic susceptibility of CsMn(NCS)_3_ has a cusp at 16.8(8) K indicating the onset of antiferromagnetic order (Fig. 2b). An increase in susceptibility at low temperature is likely due to a small fraction of a hydrated impurity. Fitting the inverse susceptibility between 100 < *T* < 300 K to the Curie–Weiss law gives *θ*_CW_ = −33.6(2) K, and *C* = 3.75(2) emu K mol^−1^, which is lower than the high-spin spin only expected value of *C*_spin only_ = 4.375 emu K mol^−1^ for Mn^2+^. We did not observe any evidence of hysteresis in the isothermal magnetisation data, consistent with antiferromagnetic order. The magnetisation reaches 1.20(6) *μ*_B_ per Mn at the largest field measured (7.00(1) T), well below the spin only saturation magnetisation, *M*_sat._ = 5 *μ*_B_ per Mn, indicative of the presence of significant antiferromagnetic interactions. The ratio of the Curie–Weiss temperature to the ordering temperature is *f* = |*θ*_CW_/*T*_N_| = 2.1, suggestive of slight frustration or low-dimensionality.

Curie–Weiss fitting of the magnetic susceptibility of CsCo(NCS)_3_ between 100 < *T* < 300 K suggests predominately antiferromagnetic interactions, *θ*_CW_ = −19.7(2) K. The large Curie constant, *C* = 4.8(2) emu K mol^−1^, compared to the high-spin spin only value *C*_spin only_ = 1.875 emu K mol^−1^, indicates that the unquenched orbital moment remains significant in these compounds and the determined *θ*_CW_ therefore likely also includes the effects of the first order spin–orbit coupling. d*χ*/d*T* shows two sharp minima, suggesting that there are potentially two ordering temperatures for the compound at 6.7(1) and 8.4(1) K. The isothermal magnetisation data measured at 2 K show a hysteresis with *H*_C_ = 0.052(2) T and a remnant magnetisation *M*_rem_ = 0.400(1) *μ*_B_ per Co, suggesting a canting angle of 15.3°. The hysteresis disappears at 1.86(1) T, when *M* = 0.88(4 *μ*_B_ per Co. Above this applied magnetic field, the magnetisation steadily increases, reaching 2.00(7 *μ*_B_ per Co at 7.00(1) T, although the moment remains unsaturated, due to a combination of single-ion anisotropy and antiferromagnetic interactions.

Our bulk magnetic property measurements suggested that both CsNi(NCS)_3_ and CsCo(NCS)_3_ are weak ferromagnets (canted antiferromagnets), or more generally have uncompensated magnetic moments, with appreciable hystereses and field-induced magnetic phase transitions. Comparatively, the absence of hysteresis and a negative *θ*_CW_ for CsMn(NCS)_3_ suggests it is an antiferromagnet.

### 2.3 Neutron diffraction

To explore the magnetic properties of these post-perovskites further, we therefore carried out neutron diffraction experiments, both single crystal and powder, to determine their ground state magnetic structures. Scale-up of our initial synthetic routes produced high quality powder and single crystal samples of CsNi(NCS)_3_ and CsMn(NCS)_3_ suitable for neutron diffraction, however the two-stage synthesis of CsCo(NCS)_3_ meant we were unable to obtain either large (mm^3^) single crystals or gram-scale pure phase microcrystalline powders, precluding neutron studies for this compound.

**Fig. 3:**
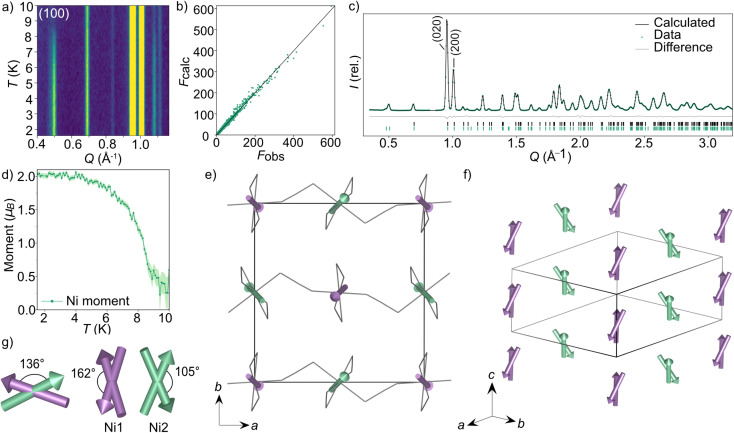
Neutron diffraction data and the magnetic structure for CsNi(NCS)_3_. (a) Thermodiffractogram measured between 1.5 and 10 K on the D1b diffractometer (ILL). The most intense magnetic Bragg reflection is indexed as the (100) planes. The *F*_obs_ against *F*_calc_ plot obtained from D19 data fit (nuclear and magnetic) collected at 1.8 K (b) and Rietveld fit obtained from D1b data fit at 1.5 K (c) for the multi pattern refinement of the *P*2_1_/*c* magnetic model describing the magnetic structure of CsNi(NCS)_3_. (d) The magnetic moment of the Ni^2+^ ions as a function of temperature obtained by Rietveld refinements of the data collected at each temperature point between 1.5 and 10 K. (e) The magnetic structure of CsNi(NCS)_3_ viewed down the *c* axis. The two unique magnetic vectors are represented with purple arrows (Ni1) and green arrows (Ni2). The Cs^+^ cations have been omitted for clarity and the thiocyanate ligands are represented as a wire frame. (f) The magnetic structure viewed down the [111] direction. (g) Angles describing the non-collinearity of the moments.

We carried out single crystal neutron diffraction measurements of CsNi(NCS)_3_ (1.8 × 0.9 × 0.3 mm^3^) using the D19 diffractometer at the Institut Laue Langevin (ILL). A low temperature data collection at 2 K, below the ordering temperature of 8.5 K, allowed us to determine the propagation vector to be ***k*** = (0, 0, 0) by indexing of the magnetic Bragg reflections. We combined these single crystal neutron diffraction (SCND) data with additional powder neutron diffraction (PND) data collected on a polycrystalline sample (1.1 g) using the powder neutron diffractometer D1b (ILL). The magnetic space groups with maximal symmetry which permit magnetic moments to exist on the two Ni^2+^ ions were determined using the Bilbao Crystallographic Server^50–52^ to be 
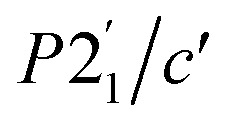
 and *P*2_1_/*c* (BNS notation).^51^ Both of these models have the same unit cell as the nuclear structure. We then refined models in each space group against the combined PND and SCND data with a multi-pattern refinement and found that only *P*2_1_/*c* was able to model the additional intensity arising from the magnetic reflections. This refined structure shows weak ferromagnetic order with two unique magnetic sites, corresponding to the two crystallographic Ni^2+^ ions (Ni1 and Ni2) in the nuclear structure. The two moments have a magnitude of 2.01(3 *μ*_B_ and were constrained to refine together with a negative correlation. The moments are directed predominately along the *c* axis with the canting only present along the *b* axis. The Ni1 moments (purple arrows Fig. 3) are canted at an angle of 162° along the −*b* direction, whilst the Ni2 moments (green arrows Fig. 3) are canted at an angle of 105° in the +*b* direction. The asymmetric canting of the two sublattices results in a net magnetisation of 0.116 *μ*_B_ per Ni along the *b* axis (+*b* direction). This is equivalent to a single Ni^2+^ canting at an angle of 6.7°, which is in agreement with the magnetometry data. The magnetic moment of Ni1 and Ni2 present a relative tilt of 136° between them.

We found that when subtracting the 2 K PND data from the 10 K data, there are some nuclear peaks which do not directly overlap at the two temperatures. This is particularly evident in the (020) and (200) reflections, which are the most intense in the diffraction patterns (Fig. 3c). On heating from 2 to 10 K, the *a* axis increases by 0.028%, whereas the *b* axis decreases in length by 0.019%. In contrast, the *c* axis remains constant within this temperature range (ESI Fig. 6†).

**Fig. 4:**
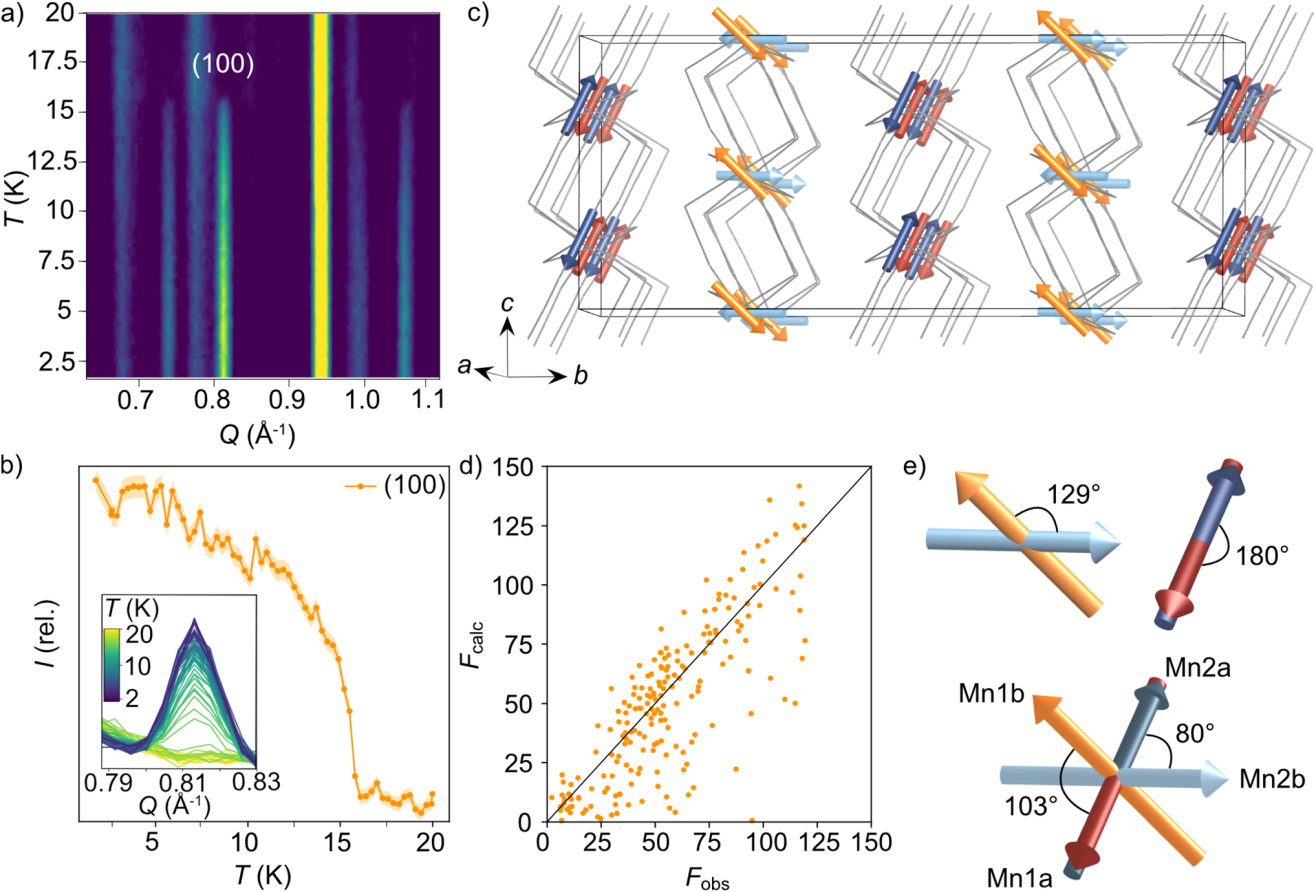
Neutron diffraction data used to determine the magnetic structure for CsMn(NCS)_3_. (a) Thermodiffractogram measured between 1.5 and 20 K on the D1b diffractometer (ILL). The most intense magnetic Bragg reflection is indexed as the (100) planes. (b) The integrated intensity of the (100) magnetic reflection at 0.81 Å^−1^ as a function of temperature. (c) The magnetic structure of CsMn(NCS)_3_. The magnetic vectors are depicted with red and orange arrows for Mn1 (red = Mn1a and orange = Mn1b) and blue arrows for Mn2 (dark = Mn2a and light = Mn2b). The Cs^+^ cations have been omitted for clarity and the thiocyanate ligands are represented as a wire frame. (d) *F*_obs_ against *F*_calc_ for the refinement of the *P*_*S*_1̄ magnetic model using the magnetic reflections observed at 2 K with the D19 diffractometer (ILL). (e) The angles between the magnetic vectors.

We were also able to measure a large single crystal of CsMn(NCS)_3_ (6.3 × 2.5 × 1.1 mm^3^) using D19 (ILL). We found in our diffraction data collected at 2 K, below *T*_N_ = 16.8 K, a number of additional Bragg reflections (2938 unique reflections) not present in our data collected at 20 K, which could not be indexed to the nuclear structure. We were able to index these additional magnetic Bragg reflections with 
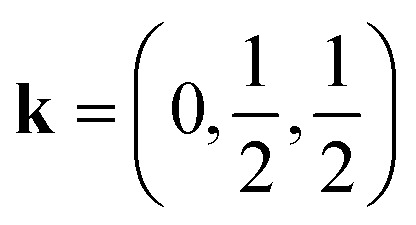
. Using the Bilbao Crystallographic Server we determined the possible magnetic space groups for this propagation vector to be *P*_*S*_1̄ and *P*_*S*_1. We solved the magnetic structures in both magnetic space groups, with the *P*_*S*_1̄ better fitting the experimental data. The model comprises of four unique magnetic sites: Mn1a and Mn1b, which arise from nuclear site Mn1 but are in alternate layers (red and orange arrows Fig. 4c), and Mn2a and Mn2b from nuclear site Mn2 (dark and light blue arrows Fig. 4c). The magnetic unit cell is related to the nuclear cell as follows *a*_mag_ = *a*_nuc_, *b*_mag_ = 2*b*_nuc_ and *c*_mag_ = 2*c*_nuc_. The magnitude of the moments for all Mn sites is 4.63(9 *μ*_B_ and were constrained to refine with a single moment value. We found that allowing the moments to refine freely did not significantly improve our fit (constrained refinement, *χ*^2^ = 68.5 compared to free refinement, *χ*^2^ = 56.7), and led to unphysically small moment sizes and unstable moment angles. Constraining the moment angles of Mn1b and Mn2b to be collinear (while allowing Mn1a and Mn2a to be non-collinear) gave *χ*^2^ = 120.3, whilst constraining all four moments to be collinear gave *χ*^2^ = 807.6. In the determined model, each of the four magnetic sublattices, derived from a unique Mn^2+^ site, order antiferromagnetically as expected from the bulk antiferromagnetic order observed in the magnetisation data. Mn1a and Mn2a moments, are aligned antiparallel within the anionic layer, while Mn1b and Mn2b are at an angle of 103° relative to each other (Fig. 4e). Powder neutron diffraction data were collected on the D1b diffractometer, which clearly shows the additional magnetic Bragg reflections. However, the limited data quality prevented quantitative refinement of these data.

### 2.4 Density functional theory calculations

**Fig. 5:**
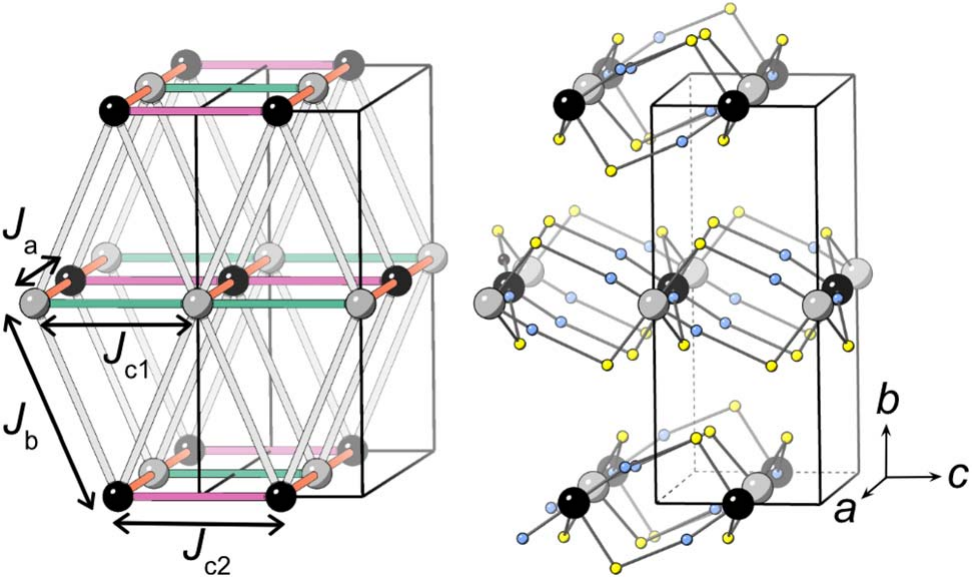
The four nearest-neighbour magnetic interactions in CsM(NCS)_3_. M1 and M2 are grey and black respectively and the thiocyanate ligands are represented with sulfur (yellow) and nitrogen (blue) atoms only. Caesium cations and carbon atoms have been removed for clarity.

Our neutron analysis provided the ground states, but the energetics which yield these states remained opaque. We therefore turned to DFT calculations. In order to correct for the typical delocalisation errors encountered in DFT, a Hubbard *U* was incorporated.^38,53^ There are four nearest-neighbour interactions (Fig. 5): *J*_a_, through the M1–NCS–M2 corner-sharing bridge; *J*_c1_, through the M1–NCS–M1 edge-sharing chain; *J*_c2_, through the M2–NCS–M2 edge-sharing chain; and *J*_b_, between the layers. The magnetic lattice therefore consists of rectangular lattices in which there are two different kinds of chain along one direction, which are then coupled together by *J*_b_. Due to the offset of the layers, this means that if *J*_a_, *J*_c1_ and *J*_c2_ are antiferromagnetic, we would expect this lattice to be frustrated. To calculate each of these interactions we therefore constructed eight 2 × 1 × 1 supercells with distinct ordering patterns (ESI Table 1†), and fitted their DFT+*U* energies to the following Hamiltonian:1
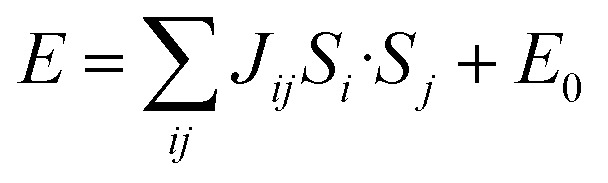
where *J*_*ij*_ denotes the superexchange interaction for the atom pair *ij* (*i.e.* each interaction is counted once), |*S*| = 1, and *E*_0_ is the energy of a hypothetical non-magnetic state. We found that for CsNi(NCS)_3_ and CsMn(NCS)_3_ we could obtain self-consistent results, but we were unable to achieve acceptable self-consistency for CsCo(NCS)_3_. The residual unquenched orbital moment for the ^4^*T*_1_ ground state is likely responsible for this and suggests that higher level calculations are likely to be required to appropriately capture the magnetic behaviour of this compound.

Our calculations indicate that all four interactions are antiferromagnetic for CsMn(NCS)_3_, with the interactions through the edge-sharing chains (*J*_c1_ and *J*_c2_) stronger than the corner-sharing bridge (*J*_a_) between them (Table 2). The magnitude of the *J*_b_ interaction is much smaller than the error and is thus not meaningful. We found that CsNi(NCS)_3_ has ferromagnetic interactions within the edge-sharing chains, with the *J*_c2_ chain notably stronger, and the interactions between chains are antiferromagnetic. As with CsMn(NCS)_3_, the *J*_b_ interaction is small and zero within error. From these interactions we were able to calculate Curie–Weiss temperatures: for Mn *θ*_CW,calc._ = −22.3(5) K and for Ni *J*_CW,calc._ = −0.05(65) K which are broadly comparable with those found experimentally. The ground states predicted by DFT are largely consistent with those determined experimentally, allowing for the fact that these non-relativistic calculations cannot predict spin-canting.

**Table 2:** DFT-derived superexchange energy, *J*_*x*_, as defined in eqn (1) for CsM(NCS)_3_, M = Ni, Mn

M^2+^	*J*_a_ a	*J*_b_ a	*J*_c1_ a	*J*_c2_ a
Ni	0.19(4)	−0.02(10)	−0.13(7)	−0.25(7)
Mn	0.78(3)	0.03(6)	2.40(6)	1.79(6)

a (meV).

## 3 Discussion

The CsM(NCS)_3_ compounds all crystallise with the post-perovskite structure. However, unlike most atomic analogues, these thiocyanate compounds adopt the structure at ambient pressure. A common characteristic of atomic post-perovskites is the presence of large octahedral tilt angles in the corresponding perovskite phase, which leaves them more susceptible to undergo the post-perovskite phase transition.^11^ Thiocyanate perovskites are already very tilted, due to the shape of the frontier bonding orbitals, which could explain the ease for formation of this structure-type for CsM(NCS)_3_.^38^ Defining the plane as the *ac* axes, the tilting occurring in these thiocyanate compounds along the edge- and corner-sharing directions can be compared. The corner-sharing octahedra, along the *a* axis, have smaller deviations away from the *ac* plane, with angles of ∠*ac*–M–S = 42(1)° and ∠*ac*–M–N = 9(1)° (ESI Fig. 9†). Along the *c* axis, the edge-sharing octahedra adopt greater tilting angles as an inherent consequence of having two thiocyanate ligands bridging each pair of metal centres in this direction. The deviation away from the *ac* plane is ∠*ac*–M–S = 58(1)° and ∠*ac*–M–N = 36(1)°. The incorporation of a molecular ligand permits access to this greater degree of tilting without the need for external pressure. This is evident from our DFT calculations which show that for these CsM(NCS)_3_ compounds the post-perovskite structure-type is lower energy than other reported thiocyanate perovskite-type structures (Table 1).

Our magnetometry and neutron diffraction measurements show that the compounds magnetically order between 6 and 16 K, significantly lower than the closest chemical analogues, the binary thiocyanates M(NCS)_2_ M = Mn, Fe, Co, Ni, Cu,^37,38^ which order at *T*_N_ = 29 K for Mn(NCS)_2_, *T*_N_ = 20 K for Co(NCS)_2_^37,42^ and *T*_N_ = 54 K for Ni(NCS)_2_.^37,41^ The atomic post-perovskites have a range in ordering temperatures, with the fluorides ordering at similar temperatures to CsM(NCS)_3_, for example post-perovskite NaNiF_3_ orders at *T*_N_ = 22 K (compared to *T*_C_ = 156 K for the perovskite phase).^12^ The reported ordering temperatures of the oxide post-perovskites are an order of magnitude larger, for example CaIrO_3_ has *T*_N_ = 115 K,^8,54,55^ likely as the oxides lie closer to the metal-insulator boundary.

One key difference between CsM(NCS)_3_ and M(NCS)_2_ is that the post-perovskites only have three-atom connections between transition metals (*μ*_13_NCS coordination mode), whereas M(NCS)_2_ have both one-atom and three-atom connections (*μ*_133_NCS). The additional M–S–M superexchange pathway in the binary thiocyanates likely strengthens the magnetic interactions in the binary thiocyanates, although DFT calculations of Cu(NCS)_2_ suggest that interactions through the M–S–C–N–M can be as strong or stronger than through M–S–M bridges.^38^ Our DFT calculations further support this, showing appreciable superexchange through the end-to-end bridging thiocyanates.

In contrast to these compounds, dca^−^ based post-perovskites containing magnetic ions do not appear to order.^24,25,36^ The transition metals in these compounds are separated by six bonds and *d*(Mn-NCNCN-Mn) = 8.9825(4) Å ([Ph_4_P]Mn(dca)_3_),^24^ compared to four bonds and *d*(Mn–NCS–Mn) = 6.37315(5) Å (CsMn(NCS)_3_). This likely reduces the superexchange further. However, Cr[Bi(SCN)_6_], with even longer superexchange pathways does order at *T*_N_ = 4.0 K,^39^ indicating that orbital overlap and orbital energy matching are also playing a key role in this.

CsM(NCS)_3_, M = Ni, Mn, Co, all adopt non-collinear magnetic structures. Non-collinearity also appears to be typical in the atomic perovskites. The only previous experimentally reported magnetic structure of a post-perovskite is of CaIrO_3_, which used magnetic resonant X-ray scattering to reveal a canted stripe antiferromagnetic ground state.^16^ Octahedral tilting is predicted to be a key parameter in determining the degree of non-collinearity,^56^ so it is expected that the post-perovskite structures are sensitive to this factor as well. Analysis of our isothermal magnetisation data for CsNi(NCS)_3_ (Fig. 2d), assuming that there is only a single magnetic site (*i.e.* only two distinct spin orientations), gives a canting angle of 6°. However our magnetic structure has two magnetic sites (*i.e.* four spin orientations), and so there are in fact three ‘canting angles’, all of which are larger than 6° (9°, 22°, and 38°). The symmetry constraints of the *P*2_1_/*c* magnetic space group means that for each pair of canted moments (*i.e.* a single magnetic site), the components of the magnetic moments along the *a* and *c* axes will be of equal magnitude and so the uncompensated magnetisation lies only along the *b* axis. In CsNi(NCS)_3_, the uncompensated moments from each magnetic site have opposite signs: +0.80 *μ*_B_ per Ni2 and −0.57 *μ*_B_ per Ni1, with a net moment of +0.114 *μ*_B_. Using bulk measurements for materials with complex magnetic structures can therefore lead to underestimates of the degree of non-collinearity.

The magnetic structure of CsMn(NCS)_3_, unlike the nickel and cobalt analogues, orders as an antiferromagnet. The neutron data reveal a 
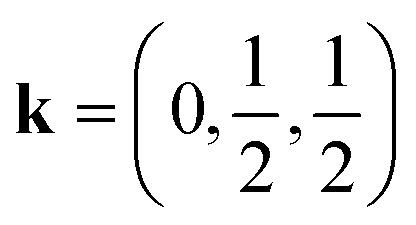
 propagation vector, which leads to four unique sublattices. As a result of the anticentring translation in the *P*_*S*_1̄ magnetic space group, each sublattice, and therefore the overall structure, is antiferromagnetic. There are two distinct kinds of layer within the magnetic structure, ‘A’ and ‘B’. Layer A, containing Mn1a and Mn2a, is antiferromagnetically correlated; but Mn1b and Mn2b in layer B are non-collinear both with respect to each other and also to Mn1a and Mn2a. The complexity of this structure is perhaps surprising, considering the relative simplicity of the nuclear structure and the lack of spin–orbit coupling expected for high spin Mn^2+^. The layers stack so that each consecutive layer is offset by *c*_nuc_/2, resulting in a triangular relationship between the interlayer moments (Fig. 5). This layered stacking pattern may generate frustration, which could explain the observed non-collinear structure.

## 4 Conclusion

In this paper, we have reported the synthesis, structure, magnetometry, single crystal and powder neutron diffraction data for three isomorphic post-perovskite thiocyanate frameworks, CsM(NCS)_3_ M = Ni, Mn, Co. Our magnetic susceptibility measurements show that all the materials magnetically order, CsNi(NCS)_3_*T*_C_ = 8.5(1) K, CsMn(NCS)_3_*T*_N_ = 16.8(8) K and CsCo(NCS)_3_*T*_C_ = 6.7(1) K. Our neutron diffraction experiments on CsNi(NCS)_3_ and CsMn(NCS)_3_ revealed both compounds have complex non-collinear ordering.

CsNi(NCS)_3_ orders as a weak ferromagnet with two magnetically distinct nickel moments. CsMn(NCS)_3_, on the other hand, orders as an antiferromagnet with a magnetic unit cell which is doubled along the nuclear *b* and *c* axes, and has four unique sublattices.

Our neutron diffraction studies have shown that despite the relative simplicity of the chemical structures, these thiocyanate post-perovskites are a rich source of unusual magnetic orderings which cannot be recognised through magnetometry data alone. Introducing molecular ligands into framework structures may therefore provide a host of unexpected and complex spin textures, motivating both future synthetic and neutron diffraction investigations.

## Data availability

Research data and analysis notebooks are available at the Nottingham Research Data Management Repository DOI: https://doi.org/10.17639/nott.7259. Raw data sets from ILL experiments can be accessed *via* links provided in references.

## Author contributions

M. G. and M. J. C. synthesised the samples; carried out the magnetic measurements and the single crystal X-ray experiments; M. G., L. C. D. and M. J. C. carried out the single crystal X-ray analysis; M. G., J. Y. L., O. F., L. C. D. and M. J. C. carried out the neutron diffraction experiments; M. G., O. F., L. C. D. and M. J. C. carried out the neutron diffraction and magnetic property analysis; S. L. carried out the density functional calculations; M. G., L. C. D. and M. J. C. wrote the paper with contributions from all the authors.

## Conflicts of interest

No conflicts of interest to declare.

## Supplementary Material

SC-014-D2SC06861C-s001

SC-014-D2SC06861C-s002
