# Supplementary material for: Non-collinear magnetism in the post-perovskite thiocyanate frameworks CsM(NCS)3
Source: Chem Sci. 2023 Feb 21;14(13):3531–40. doi: 10.1039/d2sc06861c (PMC10055978; doi:10.1039/d2sc06861c)
Supplement: SC-014-D2SC06861C-s001 [file SC-014-D2SC06861C-s001.pdf]

Electronic Supplementary Material (ESI) for Chemical Science.
This journal is © The Royal Society of Chemistry 2023

Non-collinear magnetism in the post-perovskite thiocyanate frameworks CsM(NCS)$_3$

Madeleine Geers[1, 2], Jie Yie Lee[1, 2], Sanliang Ling[3], Oscar Fabelo[2], Laura Cañadillas-Delgado[2], and Matthew J. Cliffe *[1]

[1]School of Chemistry, University Park, Nottingham, NG7 2RD, United Kingdom

[2]Institut Laue-Langevin, 71 avenue des Martyrs CS 20156, 38042 Grenoble Cedex 9, France

[3]Advanced Materials Research Group, Faculty of Engineering, University of Nottingham, University Park, Nottingham NG7 2RD, United Kingdom

February 2, 2023

Contents

List of Figures 1

List of Tables 2

1 Experimental 3

1.1 Synthesis of CsM(NCS)$_3$ 3

1.2 Single Crystal X-ray Diffraction 3

1.3 Single Crystal Neutron Diffraction 3

1.4 Powder Neutron Diffraction 3

1.5 Magnetic Measurements 4

1.6 Density Functional Theory Calculations 4

List of Figures

1 Variable temperature magnetic susceptibility product measured over the range 2–300 K (zero-field cooled). The high temperature spin only Curie value, C, is indicated with a dashed line for a) CsNi(NCS)$_3$ ($C = 1.0$ emu K mol^{-1}); b) CsMn(NCS)$_3$ ($C = 4.375$ emu K mol^{-1}); c) CsCo(NCS)$_3$ ($C = 1.875$ emu K mol^{-1}). 5

2 Inverse of magnetic susceptibility (zero-field cooled) between 1.5 and 300 K. The high temperature data was modelled using the Curie-Weiss law ($180 < T < 300$ K for Ni; $100 < T < 300$ K for Co and Mn), black line. 6

3 Inverse of magnetic susceptibility for CsNi(NCS)$_3$. The high temperature data was modelled using the Curie-Weiss law for a) $100 < T < 300$ K, $\theta_{\text{CW}} = +1.5(4)$ and b) $200 < T < 300$ K, $\theta_{\text{CW}} = -12.7(8)$ K, black line. 7

*matthew.cliffe@nottingham.ac.uk

4 An expansion of the isothermal magnetisation data around the hysteresis for a) $CsNi(NCS)_3$ and b) $CsCo(NCS)_3$. 7
5 Rietveld fits (black line) for $CsNi(NCS)_3$ data (green circles) collected with the D1b diffractometer at a) 10 K and b) 1.5 K. The black markers indicate the position of nuclear Bragg reflections and green markers for magnetic Bragg reflections. Data at 0.85 Å have been omitted due to the presence of an impurity. 8
6 $CsNi(NCS)_3$ lattice parameters between 1.5 and 10 K, obtained from Rietveld refinements of neutron diffraction data measured using the D1b diffractometer (ILL). The verical dashed black line indicates the magnetic ordering temperature ($T_C = 8.5$ K). A linear fit (dark green line) was used to calculated the coefficient of thermal expansion. As the change in sin(β) is at least one of magnitude smaller than the change in the a, b and c axes, the thermal expansion tensor will not significantly rotate over this temperature range. a) a axis, $\alpha_a = 19(3)$ MK^{-1}. b) b axis, $\alpha_b = -13(3)$ MK^{-1}. c) c axis, $\alpha_c = 5(2)$ MK^{-1}. d) sin(β) , $\alpha_{sin(\beta)} = 0.3(1)$ MK^{-1}. e) Volume, $\alpha_V = 11(5)$ MK^{-1}. . . . 9
7 Other $AM(NCS)_3$ structures with perovskite-analogue structure types: a) $CsCd(NCS)_3$;[1] b) $[NH_4]_2NiCd(NCS)_6$.[2] Caesium = pink, cadmium = purple, nickel = grey, nitrogen = blue, carbon = black, sulfur = yellow, hydrogen = pale pink. 10
8 Orientation of the ordered magnetic moments for a) $CsNi(NCS)_3$ viewed down the magnetic b axis, b) $CsNi(NCS)_3$ viewed down the magnetic a axis and c) $CsMn(NCS)_3$ viewed down the magnetic a axis. 10
9 Angles between the ac plane (black line) and M$-$N and M$-$S bond angles in the direction of a) corner-sharing octahedra and b) edge-sharing octahedra in $CsM(NCS)_3$. These angels are the same (to the nearest integer) for both M1 and M2. Metal = pink, nitrogen = blue, carbon = black, sulfur = yellow. 11
10 Single crystals used in the neutron diffraction experiments for a) $CsNi(NCS)_3$ and b) $CsMn(NCS)_3$. 11

List of Tables

1 The configurations used to calculate the ground state interactions and the relative energy of each configuration for $CsM(NCS)_3$, M = Ni, Mn. Each interaction is given as a relative interaction where + is ferromagnetic, $-$ is antiferromagnetic, and 0 where the interactions are compensated. 4
2 Single crystal experimental details for $CsCo(NCS)_3$ (X-ray data) and $CsNi(NCS)_3$ (neutron data) 12
3 Single crystal experimental details for $CsMn(NCS)_3$, $2 - 20$ K (neutron data) 13
4 Single crystal experimental details for $CsMn(NCS)_3$, $50 - 300$ K (50 and 300 K neutron data, 120 K X-ray data) 14

1 Experimental

1.1 Synthesis of $CsM(NCS)_3$

Aqueous solutions of $MnSO_4 \cdot H_2O$ (254 mg, 1.5 mmol, 3 mL) and $Ba(SCN)_2 \cdot 3\,H_2O$ (461 mg, 1.5 mmol, 15 mL) were combined and stirred for 24 h. The opaque white solution was filtered, and the white residue of $BaSO_4$ removed to leave a filtrate of aqueous $Mn(NCS)_2$. Cs_2SO_4 (272 mg, 0.75 mmol) and $Ba(SCN)_2 \cdot 3\,H_2O$ (231 mg, 0.75 mmol) were dissolved in distilled water (10 mL) and added to the $Mn(NCS)_2$ solution. The mixture was stirred for 24 h, and filtered, removing a white residue of $BaSO_4$. The colourless filtrate solution of $CsMn(NCS)_3$ was concentrated under reduced pressure and left to stand for 48 h yielding pale green single crystals of $CsMn(NCS)_3$.
$CsCo(NCS)_3$ and $CsNi(NCS)_3$ were synthesised in an analogous method from $CoSO_4 \cdot 7\,H_2O$ (421 mg, 1.5 mmol) and $NiSO_4 \cdot 6\,H_2O$ (394 mg, 1.5 mmol) respectively. This yielded purple, $CsCo(NCS)_3$, and green, $CsNi(NCS)_3$, microcrystalline powders. Single crystals of these compounds were obtained by slow evaporation recrystallisations from concentrated aqueous solutions. The synthesis of $CsNi(NCS)_3$ has been previously reported through the reaction of $NiCl_2 \cdot 6\,H_2O$, Cs_2CO_3 and NH_4SCN.[3]

1.2 Single Crystal X-ray Diffraction

Single crystals were selected and mounted using Fomblin® (YR-1800 perfluoropolyether oil) on a polymer-tipped MiTeGen MicroMountTM and cooled rapidly to 120 K in a stream of cold N_2 using an Oxford Cryosystems open flow cryostat.[4] Single crystal X-ray diffraction data were collected on an Oxford Diffraction GV1000 (AtlasS2 CCD area detector, mirror-monochromated Cu-Kα radiation source; $\lambda = 1.541$ Å; ω scans). Cell parameters were refined from the observed positions of all strong reflections and absorption corrections were applied using a Gaussian numerical method with beam profile correction (CrysAlisPro).[5] The structure was solved within Olex2[6] by dual space iterative methods (SHELXT),[7] least squares refinement of the structural model was carried using (SHELXL).[8] Structures were checked with checkCIF (https://checkcif.iucr.org).

1.3 Single Crystal Neutron Diffraction

Monochromatic single crystal neutron diffraction data for $CsMn(NCS)_3$ and $CsNi(NCS)_3$ were collected on the four-circle D19 diffractometer at the Institut Laue Langevin (ILL) Grenoble, France, using neutrons with a wavelength of 1.455 Å ($CsMn(NCS)_3$) and 1.457 Å ($CsNi(NCS)_3$) provided by a flat Cu monochromator using the 220 reflection at $2\theta_M = 69.91°$ take-off angle. The samples were each placed in a closed-circuit displex cooling device, which was operated following a ramp of 2 K min^{-1}.
NOMAD software from the ILL was used for data collection. Unit cell determinations were performed using PFIND and DIRAX programs, and processing of the raw data was applied using RETREAT, RAFD19 and Int3D programs.[9–12] For $CsNi(NCS)_3$, the data were corrected for the absorption of the low-temperature device using the D19ABS program[13] and for the size and composition of the crystal. For $CsMn(NCS)_3$ the data were corrected for the absorption of the low-temperature device using the D19ABS program. Structural models were solved using the *SUPERFLIP* program and refined using Jana2006.[14,15]

1.4 Powder Neutron Diffraction

Constant wavelength powder neutron diffraction data for $CsMn(NCS)_3$ and $CsNi(NCS)_3$ were collected on the high-intensity medium resolution D1b diffractometer[16] at ILL, France. The incident wavelength was $\lambda = 2.52$ Å and the scattering was measured over an angular range of $2 < 2\theta < 128°$. Thermal diffractograms for $CsMn(NCS)_3$ were collected between 1.5 K and 20 K heated with a programmed ramp of 0.06 K min^{-1}. Long acquisition measurements were collected at 1.5 K and 20 K.

Thermal diffractograms for $CsNi(NCS)_3$ were collected between 1.5 K and 10 K heated with a programmed ramp of 0.025 K min^{-1}. Long acquisition measurements were collected at 1.5 K and 10 K. NOMAD software from the ILL was used for data collection. Refinements of the magnetic model were completed using the FullProf program.[17]

1.5 Magnetic Measurements

Measurements of the magnetic susceptibility were carried out on samples of $CsNi(NCS)_3$ (34.5 mg) and $CsCo(NCS)_3$ (14.3 mg) using a Quantum Design Magnetic Property Measurements System (MPMS) 3 Superconducting Quantum Interference Device (SQUID) magnetometer, and for $CsMn(NCS)_3$ (9.6 mg) using an MPMS XL. The zero-field-cooled (ZFC) and field-cooled (FC) susceptibility was measured in an applied field of 0.01 T over the temperature range 2–300 K. As *M*(H) is linear in this field, the small-field approximation for the susceptibility, $\chi(T) \simeq \frac{M}{H}$, where *M* is the magnetisation and *H* is the magnetic field intensity, was taken to be valid.
Isothermal magnetisation measurements were carried out at 2 K over the field range -7 to $+7$ T for $CsMn(NCS)_3$ and $CsCo(NCS)_3$, and a range of -6 to $+6$ T for $CsNi(NCS)_3$. Data were corrected for diamagnetism of the sample using Pascal's constants.[18]

1.6 Density Functional Theory Calculations

We have performed density functional theory (DFT) calculations to probe the structures and energetics of spin order of the compounds considered in this study. The spin-polarised DFT+*U* method (with Grimme's D3 van der Waals correction)[19] was employed in structural optimisations and energy calculations, using the Vienna Ab initio Simulation Package (VASP 5.4.4).[20] In our DFT+*U* calculations, we used *U* values of 3.6, 5.0 and 5.1 eV for d-electrons of Mn^{2+}, Co^{2+} and Ni^{2+} cations,[21,22] respectively, and a range of ferromagnetic and anti-ferromagnetic spin solutions were considered for divalent magnetic cations (Table 1). We used a plane-wave basis set with a kinetic energy cutoff of 520 eV to expand the wave functions. The Perdew-Burke-Ernzerhof functional[23] in combination with the projector augmented wave method[24,25] were used to solve the Kohn-Sham equations. An energy convergence threshold of 10^{-4} eV was used for all total energy calculations, and the structural optimisations, including cell parameters and atomic positions, were considered converged if all interatomic forces fall below 0.01 eV Å^{-1}. All DFT calculations have been performed in the $2 \times 1 \times 1$ supercell (8 formula units per cell) using a $6 \times 5 \times 5$ k-grid (which corresponds to a k-points spacing of around 0.1 Å^{-1}).

Table 1: The configurations used to calculate the ground state interactions and the relative energy of each configuration for $CsM(NCS)_3$, M = Ni, Mn. Each interaction is given as a relative interaction where + is ferromagnetic, $-$ is antiferromagnetic, and 0 where the interactions are compensated.

Structure	J_a	J_b	J_{c1}	J_{c2}	E($CsNi(NCS)_3$) *	E($CsMn(NCS)_3$) *
FM	+	+	+	+	0.000	0.000
AFM1	+	$-$	+	+	-1.184	-3.349
AFM2	$-$	+	+	+	-0.212	-0.121
AFM3	0	$-$	$-$	$-$	-0.041	-11.511
AFM4	0	$-$	$-$	$-$	-0.171	-11.587
AFM5	0	+	$-$	$-$	0.389	-8.669
AFM6	$-$	$-$	+	+	-0.914	-3.330
AFM7	0	0	+	$-$	-0.238	-6.643
AFM8	0	0	$-$	+	$+0.014$	-5.425

* meV per formula unit

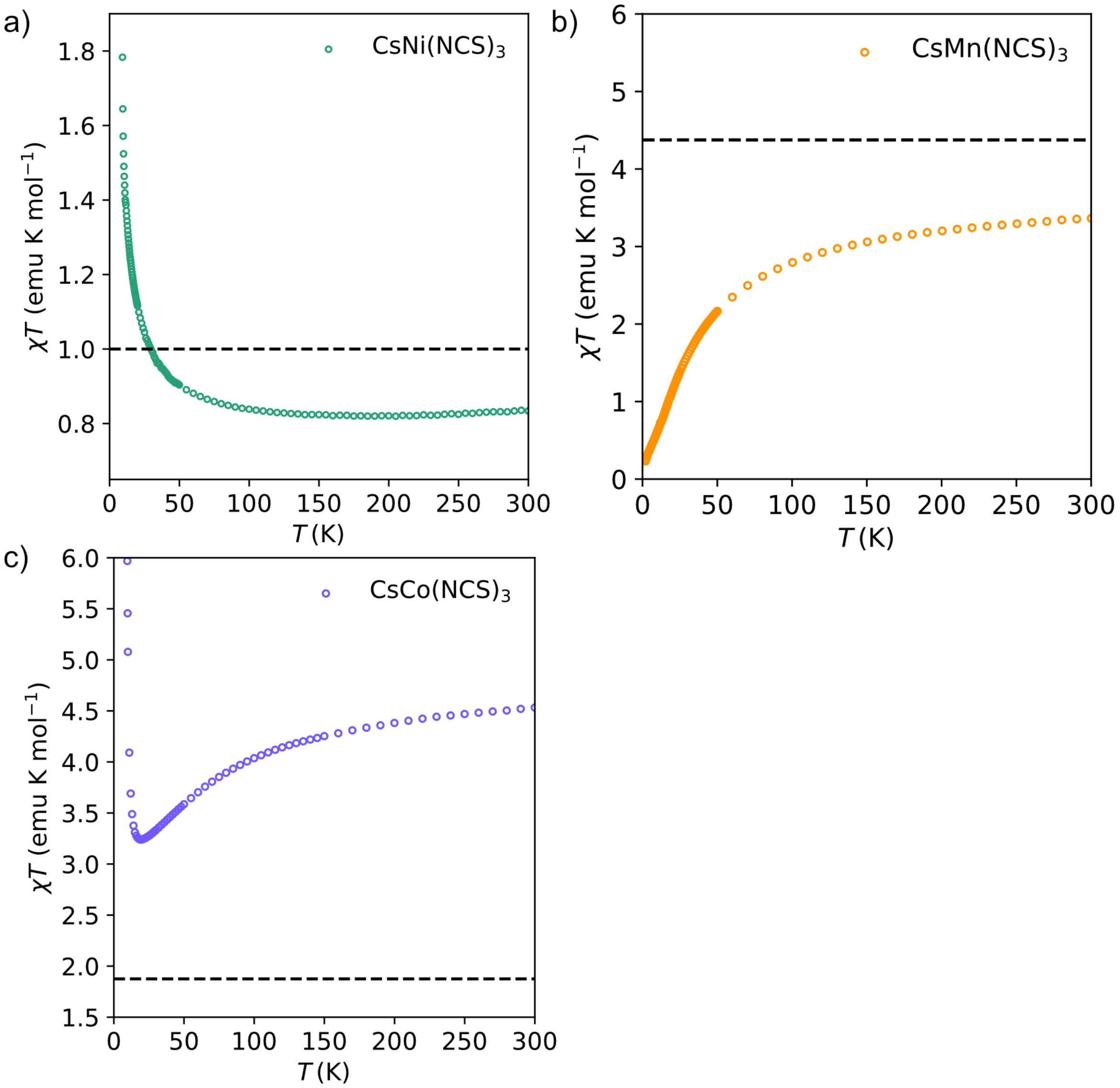

Figure 1: Variable temperature magnetic susceptibility product measured over the range 2–300 K (zero-field cooled). The high temperature spin only Curie value, C, is indicated with a dashed line for a) $CsNi(NCS)_3$ ($C = 1.0$ emu K mol^{-1}); b) $CsMn(NCS)_3$ ($C = 4.375$ emu K mol^{-1}); c) $CsCo(NCS)_3$ ($C = 1.875$ emu K mol^{-1}).

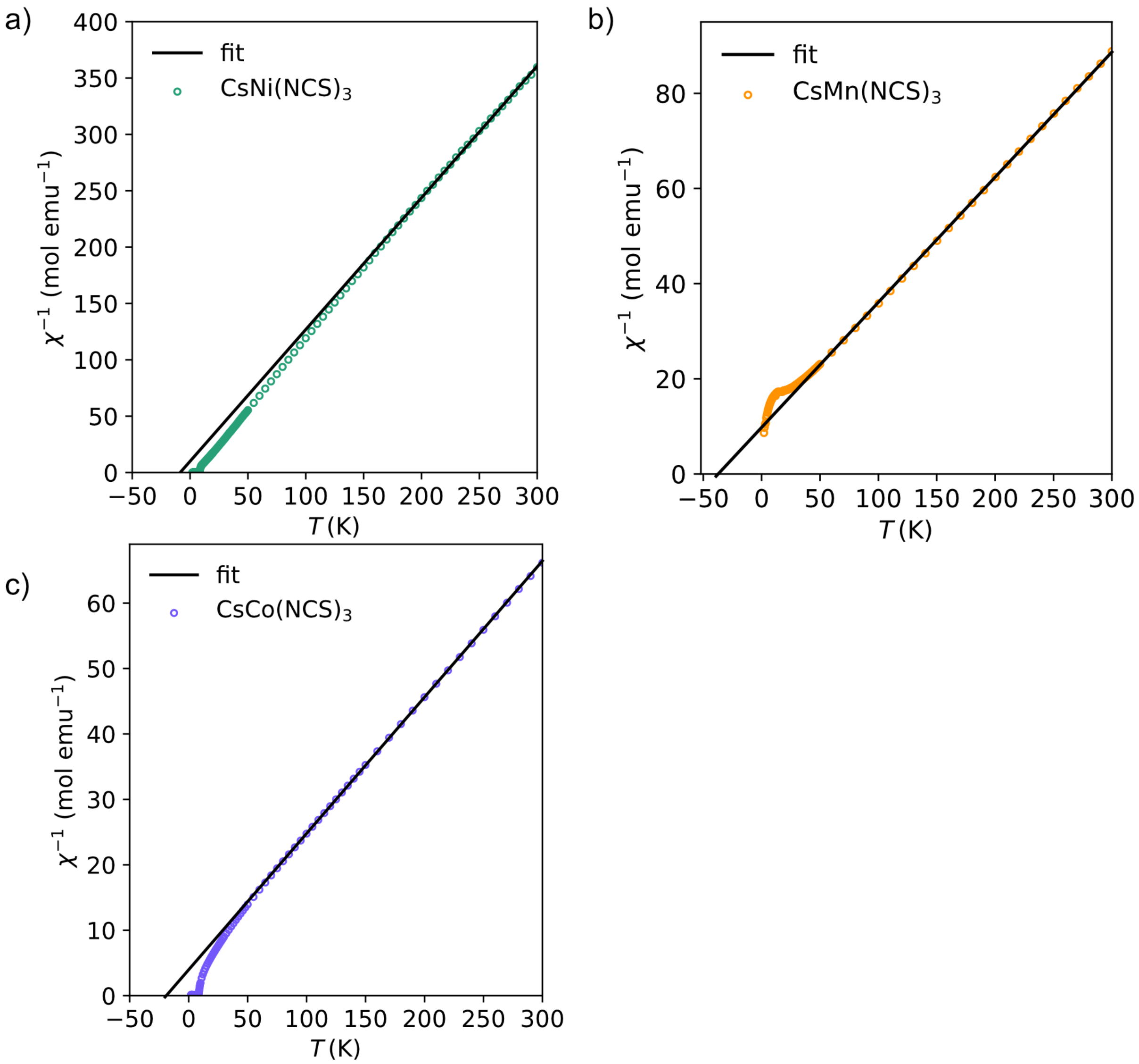

Figure 2: Inverse of magnetic susceptibility (zero-field cooled) between 1.5 and 300 K. The high temperature data was modelled using the Curie-Weiss law ($180 < T < 300$ K for Ni; $100 < T < 300$ K for Co and Mn), black line.

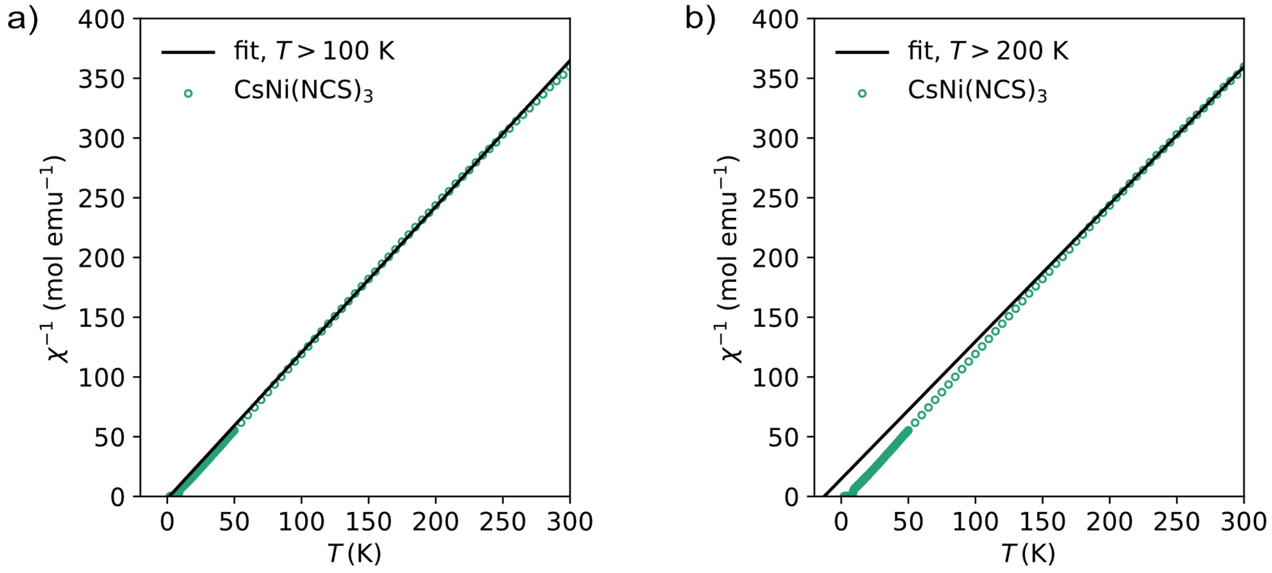

Figure 3: Inverse of magnetic susceptibility for $CsNi(NCS)_3$. The high temperature data was modelled using the Curie-Weiss law for a) $100 < T < 300$ K, $\theta_{\mathrm{CW}} = +1.5(4)$ and b) $200 < T < 300$ K, $\theta_{\mathrm{CW}} = -12.7(8)$ K, black line.

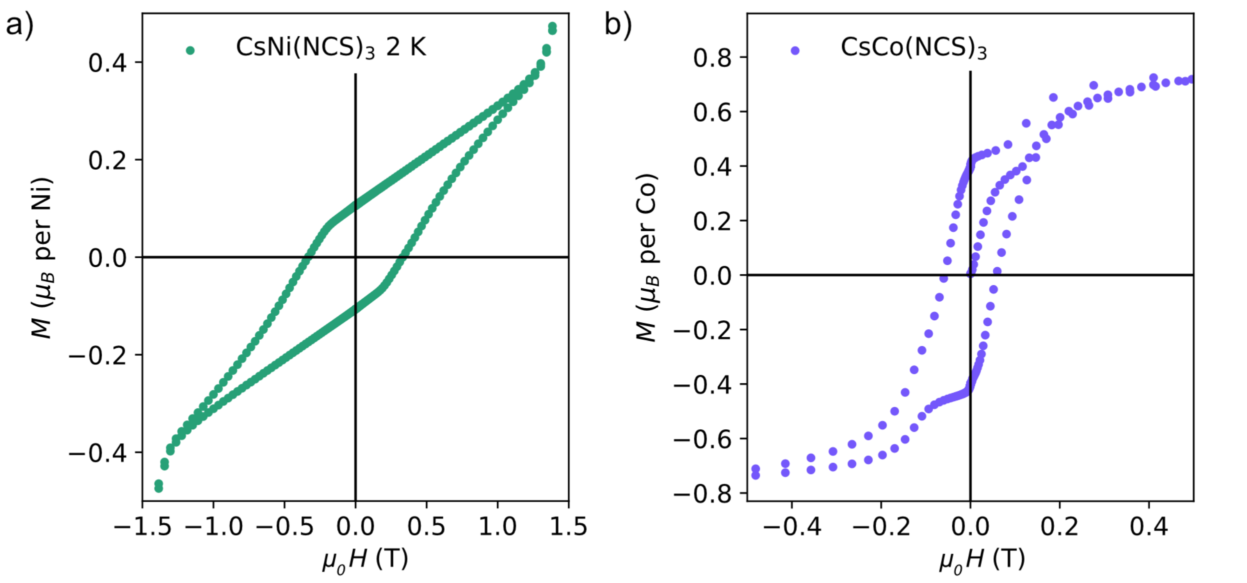

Figure 4: An expansion of the isothermal magnetisation data around the hysteresis for a) $CsNi(NCS)_3$ and b) $CsCo(NCS)_3$.

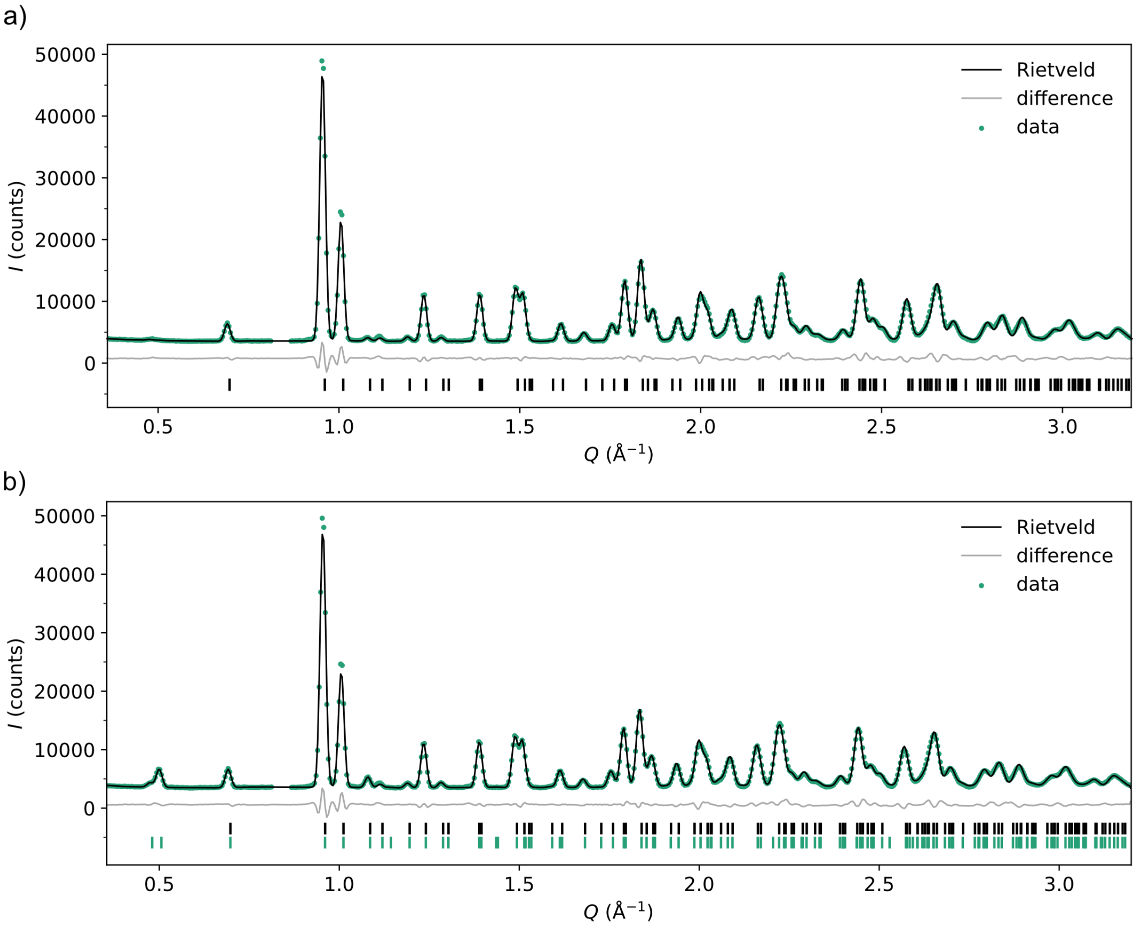

Figure 5: Rietveld fits (black line) for $CsNi(NCS)_3$ data (green circles) collected with the D1b diffractometer at a) 10 K and b) 1.5 K. The black markers indicate the position of nuclear Bragg reflections and green markers for magnetic Bragg reflections. Data at 0.85 Å have been omitted due to the presence of an impurity.

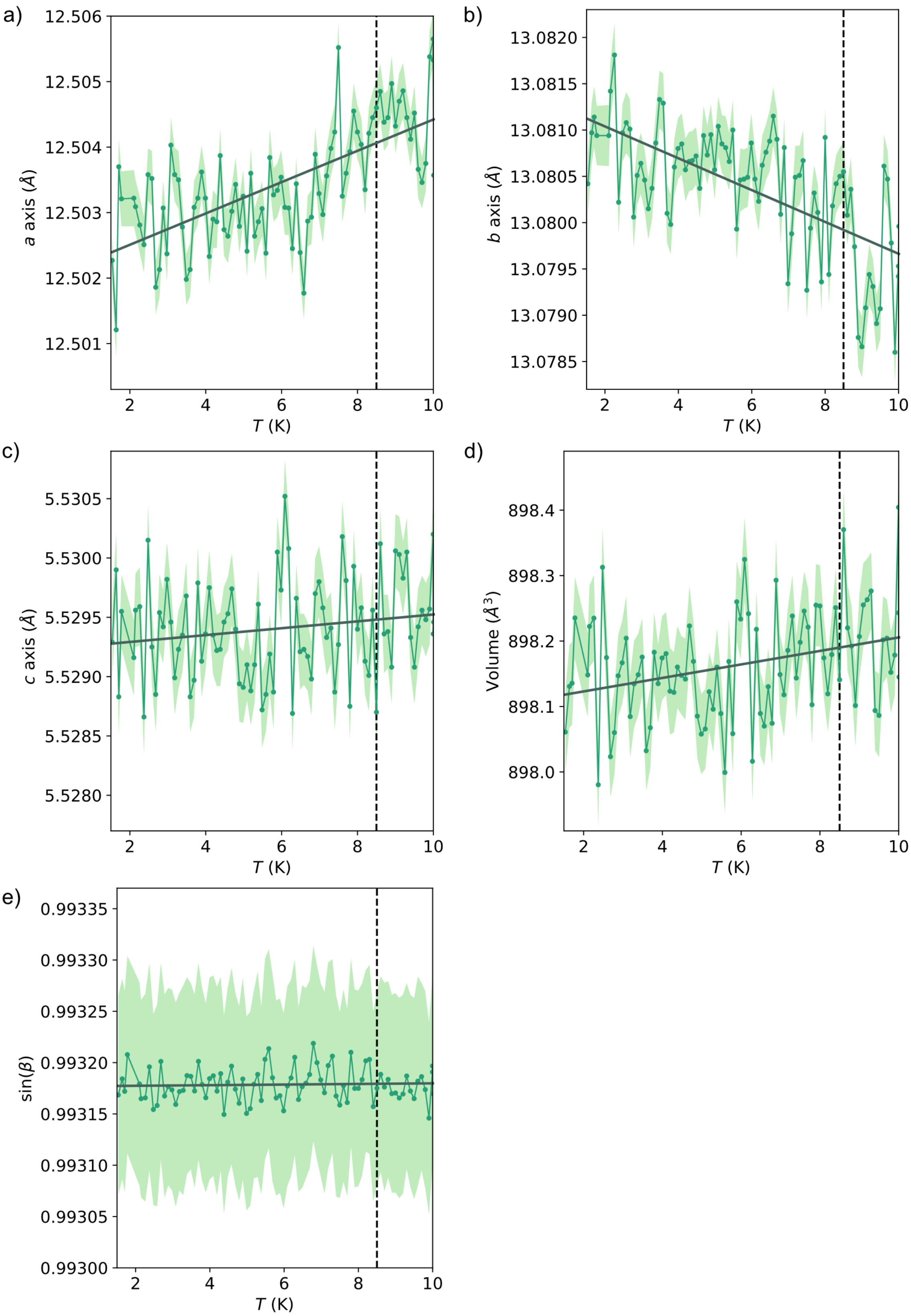

Figure 6: $CsNi(NCS)_3$ lattice parameters between 1.5 and 10 K, obtained from Rietveld refinements of neutron diffraction data measured using the D1b diffractometer (ILL). The verical dashed black line indicates the magnetic ordering temperature ($T_C = 8.5$ K). A linear fit (dark green line) was used to calculated the coefficient of thermal expansion. As the change in sin(β) is at least one of magnitude smaller than the change in the a, b and c axes, the thermal expansion tensor will not significantly rotate over this temperature range. a) a axis, $\alpha_a = 19(3)$ MK^{-1}. b) b axis, $\alpha_b = -13(3)$ MK^{-1}. c) c axis, $\alpha_c = 5(2)$ MK^{-1}. d) sin(β) , $\alpha_{sin(\beta)} = 0.3(1)$ MK^{-1}. e) Volume, $\alpha_V = 11(5)$ MK^{-1}.

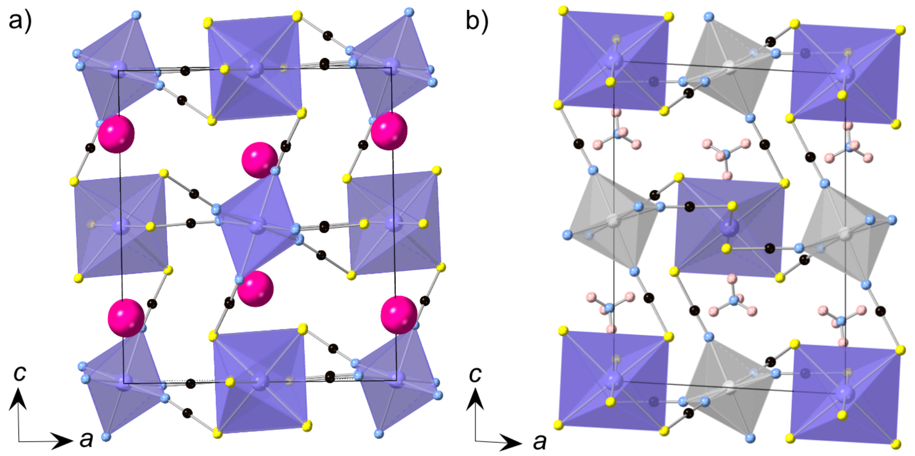

Figure 7: Other AM(NCS)$_3$ structures with perovskite-analogue structure types: a) CsCd(NCS)$_3$;[1] b) $[NH_4]_2$NiCd(NCS)$_6$.[2] Caesium = pink, cadmium = purple, nickel = grey, nitrogen = blue, carbon = black, sulfur = yellow, hydrogen = pale pink.

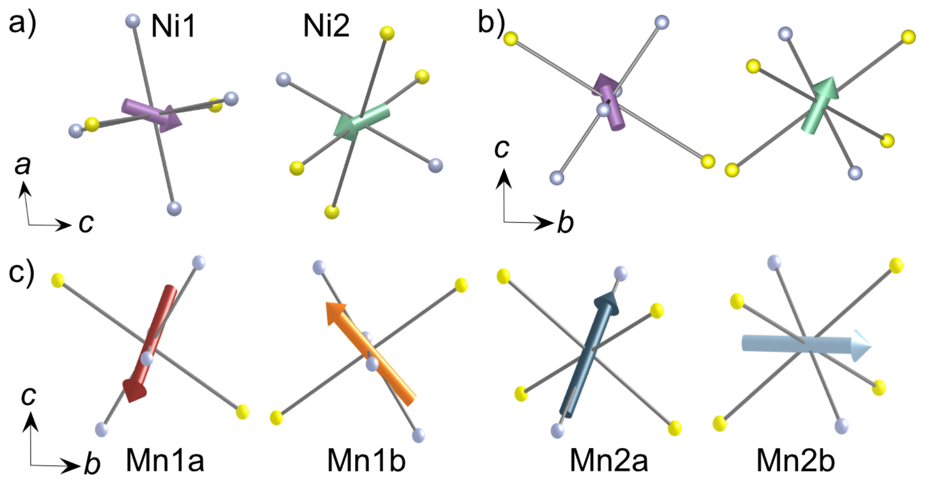

Figure 8: Orientation of the ordered magnetic moments for a) CsNi(NCS)$_3$ viewed down the magnetic b axis, b) CsNi(NCS)$_3$ viewed down the magnetic a axis and c) CsMn(NCS)$_3$ viewed down the magnetic a axis.

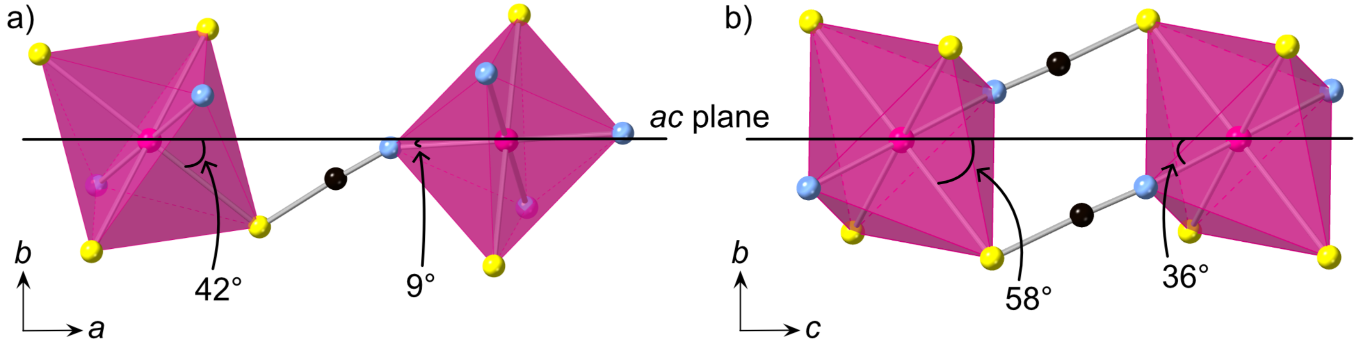

Figure 9: Angles between the ac plane (black line) and M−N and M−S bond angles in the direction of a) corner-sharing octahedra and b) edge-sharing octahedra in $CsM(NCS)_3$. These angels are the same (to the nearest integer) for both M1 and M2. Metal = pink, nitrogen = blue, carbon = black, sulfur = yellow.

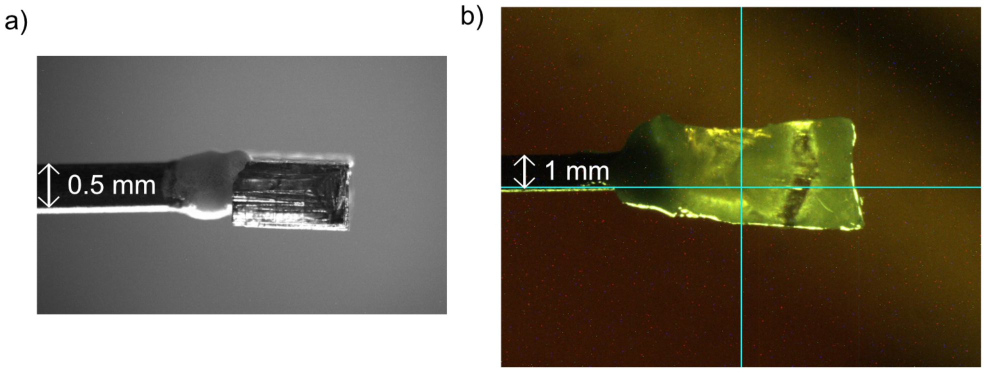

Figure 10: Single crystals used in the neutron diffraction experiments for a) $CsNi(NCS)_3$ and b) $CsMn(NCS)_3$.

Table 2: Single crystal experimental details for $CsCo(NCS)_3$ (X-ray data) and $CsNi(NCS)_3$ (neutron data)

Crystal data	Co 120K X	Ni 15K N
Chemical formula	$C_6Co_2N_6S_6 \cdot 2(Cs)$	$C_6Ni_2N_6S_6 \cdot 2(Cs)$
M_r	732.16	731.69
Temperature (K)	120	15
λ (Å)	1.54184	1.4568
Radiation type	Cu $K\alpha$	Neutron
Crystal system	Monoclinic	Monoclinic
Space group	$P2_1/n$	$P2_1/n$
a (Å)	12.6103 (1)	12.5261 (10)
b (Å)	13.2395 (2)	13.0853 (17)
c (Å)	5.5786 (1)	5.5409 (5)
β (°)	96.445 (1)	96.560 (6)
V (Å^3)	925.48 (2)	902.25 (16)
Z	2	2
μ (mm^{-1})	50.61	0.02
Crystal size (mm)	$0.13 \times 0.04 \times 0.02$	$1.8 \times 0.9 \times 0.3$ (radius= 1)
Data collection		
Diffractometer	GV1000, AtlasS2 *	D19 (ILL) **
Transmission min., max.	0.445, 0.942	$-$
No. of measured, independent and observed $[I > 2\sigma(I)]$ reflections	17041, 1847, 1809	3115, 1304, 876
R_{int}	0.051	0.077
(sin $\theta/\lambda)_{\text{max}}$ (Å^{-1})	0.620	0.598
Data completeness	0.999	0.806
Refinement		
$R[F^2 > 2\sigma\ (F^2)]$, $\omega R(F^2)$, S	0.019, 0.048, 1.04	0.114, 0.119, 3.72
No. of reflections	1847	1304
No. of parameters	103	99
$\Delta\rho >_{\text{max}}$, $\Delta\rho >_{\text{min}}$ (e Å^{-3})	+0.66, -0.87	-

* Absorption correction method: gaussian, *CrysAlis PRO* 1.171.40.53 (Rigaku Oxford Diffraction, 2019). Numerical absorption correction based on gaussian integration over a multifaceted crystal model Empirical absorption correction using spherical harmonics, implemented in SCALE3 ABSPACK scaling algorithm.

** Absorption correction was applied using D19ABS program for the low-temperature device[13] and the size and composition of the crystal.

Table 3: Single crystal experimental details for $CsMn(NCS)_3$, $2-20$ K (neutron data)

Crystal data	Mn 2K N	Mn 13K N	Mn 20K N
Chemical formula	$C_6Mn_2N_6S_6 \cdot 2\,(Cs)$	$C_6Mn_2N_6S_6 \cdot 2\,(Cs)$	$C_6Mn_2N_6S_6 \cdot 2\,(Cs)$
M_r	724.18	724.18	724.18
Temperature (K)	2	13	20
λ (Å)	1.4451	1.4451	1.4451
Radiation type	Neutron	Neutron	Neutron
Crystal system	Monoclinic	Monoclinic	Monoclinic
Space group	$P2_1/n$	$P2_1/n$	$P2_1/n$
a (Å)	12.7468 (3)	12.7463 (3)	12.7469 (3)
b (Å)	13.2446 (3)	13.2447 (3)	13.2457 (3)
c (Å)	5.6541 (1)	5.6540 (1)	5.6542 (1)
β (°)	95.8390 (16)	95.8382 (16)	95.8446 (16)
V (Å^3)	949.61 (4)	949.56 (4)	949.70 (4)
Z	2	2	2
μ (mm^{-1})	0.02	0.02	0.02
Crystal size (mm)	$6.3 \times 2.6 \times 1.1$ (radius= 3)	$6.3 \times 2.6 \times 1.1$ (radius= 3)	$6.3 \times 2.6 \times 1.1$ (radius= 3)
Data collection			
Diffractometer	D19 (ILL) *	D19 (ILL) *	D19 (ILL) *
Absorption correction	-	-	-
No. of measured, independent and observed $[I > 2\sigma(I)]$ reflections	5326, 1659, 1616	5513, 1656, 1649	5516, 1656, 1649
R_{int}	0.030	0.027	0.027
(sin $\theta/\lambda)_{max}$ (Å^{-1})	0.601	0.599	0.599
Data completeness	0.959	0.968	0.968
Refinement			
$R[F^2 > 2\sigma\ (F^2)]$, $\omega R(F^2)$, S	0.033, 0.081, 2.49	0.035, 0.097, 3.60	0.037, 0.108, 2.97
No. of reflections	1659	1656	1656
No. of parameters	103	103	103
$\Delta\rho >_{max}$, $\Delta\rho >_{min}$ (e Å^{-3})	-	-	-

*Absorption correction was applied using D19ABS program for the low-temperature device.[13]

Table 4: Single crystal experimental details for $CsMn(NCS)_3$, $50 - 300$ K (50 and 300 K neutron data, 120 K X-ray data)

Crystal data	Mn 50K N	Mn 120K X	Mn 300K N
Chemical formula	$C_6Mn_2N_6S_6 \cdot 2\,(Cs)$	$C_6Mn_2N_6S_6 \cdot 2\,(Cs)$	$C_6Mn_2N_6S_6 \cdot 2\,(Cs)$
M_r	724.18	724.18	724.18
Temperature (K)	50	120	300
λ (Å)	1.4451	1.54184	1.4451
Radiation type	Neutron	X-ray	Neutron
Crystal system	Monoclinic	Monoclinic	Monoclinic
Space group	$P2_1/n$	$P2_1/n$	$P2_1/n$
a (Å)	12.7661 (3)	12.8159 (3)	12.8136 (5)
b (Å)	13.2731 (4)	13.3648 (3)	13.4916 (7)
c (Å)	5.6588 (2)	5.6774 (1)	5.6692 (2)
β (°)	95.9037 (17)	95.999 (2)	96.346 (2)
V (Å^3)	953.77 (5)	967.11 (6)	974.06 (13)
Z	2	2	2
μ (mm^{-1})	0.02	45.62	0.02
Crystal size (mm)	$6.3 \times 2.6 \times 1.1$ (radius= 3)	$0.37 \times 0.21 \times 0.14$	$6.3 \times 2.6 \times 1.1$ (radius= 3)
Data collection			
Diffractometer	D19 (ILL) **	SuperNova, Dual, Cu at home/near, Atlas *	D19 (ILL) **
Transmission min., max.	-	0.007, 0.181	-
No. of measured, independent and observed $[I > 2\sigma(I)]$ reflections	3875, 1432, 1401	8091, 1924, 1875	2318, 1158, 1046
R_{int}	0.030	0.093	0.026
(sin $\theta/\lambda)_{\text{max}}$ (Å^{-1})	0.600	0.622	0.599
Data completeness	0.832	0.993	0.664
Refinement			
$R[F^2 > 2\sigma\ (F^2)]$, $\omega R(F^2)$, S	0.035, 0.085, 1.96	0.063, 0.160, 1.05	0.039, 0.076, 1.70
No. of reflections	1432	1924	1158
No. of parameters	103	103	103
$\Delta\rho >_{\text{max}}$, $\Delta\rho >_{\text{min}}$ (e Å^{-3})	-	+4.76, -1.57	-

*Absorption correction method: gaussian, *CrysAlis PRO* 1.171.40.53 (Rigaku Oxford Diffraction, 2019). Numerical absorption correction based on gaussian integration over a multifaceted crystal model Empirical absorption correction using spherical harmonics, implemented in SCALE3 ABSPACK scaling algorithm.

**Absorption correction was applied using D19ABS program for the low-temperature device.[13]

References

[1] G. Thiele *et al.*, *Zeitschrift fur Anorganische und Allgemeine Chemie*, 1980, **464**, 255–267.
[2] K.-P. Xie *et al.*, *CrystEngComm*, 2016, **18**, 4495–4498.
[3] M. Fleck, *Acta Crystallographica*, 2004, **C60**, i63–i65.
[4] J. Cosier *et al.*, *Journal of Applied Crystallography*, 1986, **19**, 105–107.
[5] R. O. Diffraction, *CrysAlisPro Software system, version 1.171.40.45a*, 2018.
[6] O. V. Dolomanov *et al.*, *Journal of Applied Crystallography*, 2009, **42**, 339–341.
[7] G. M. Sheldrick, *Acta Crystallographica Section A: Foundations of Crystallography*, 2015, **71**, 3–8.
[8] G. M. Sheldrick, *Acta Crystallographica Section C Structural Chemistry*, 2015, **71**, 3–8.
[9] A. J. M. Duisenberg, *Journal of Applied Crystallography*, 1992, **25**, 92–96.
[10] G. J. McIntyre *et al.*, *Acta Crystallographica Section A Foundations of Crystallography*, 1988, **44**, 257–262.
[11] C. Wilkinson *et al.*, *Journal of Applied Crystallography*, 1988, **21**, 471–478.
[12] N. A. Katcho *et al.*, *Crystals*, 2021, **11**, 897.
[13] J. C. Matthewman *et al.*, *Journal of Applied Crystallography*, 1982, **15**, 167–173.
[14] V. Petříček *et al.*, *Zeitschrift für Kristallographie-Crystalline Materials*, 2014, **5**, 345–352.
[15] V. Petříček *et al.*, *Zeitschrift für Kristallographie - Crystalline Materials*, 2016, **231**, 301–312.
[16] I. P. Orench *et al.*, *Journal of Physics: Conference Series*, 2014, **549**, 012003.
[17] J. Rodríguez-Carvajal, *Physica B: Condensed Matter*, 1993, **192**, 55–69.
[18] G. A. Bain *et al.*, *Journal of Chemical Education*, 2008, **85**, 532.
[19] S. Grimme *et al.*, *The Journal of Chemical Physics*, 2010, **132**, 154104.
[20] G. Kresse *et al.*, *Physical Review B*, 1996, **54**, 11169–11186.
[21] W. E. Pickett *et al.*, *Physical Review B*, 1998, **58**, 1201–1209.
[22] F. Zhou *et al.*, *Physical Review B*, 2004, **70**, 235121.
[23] J. P. Perdew *et al.*, *Physical Review Letters*, 1996, **77**, 3865–3868.
[24] P. E. Blöchl, *Physical Review B*, 1994, **50**, 17953–17979.
[25] D. Hobbs *et al.*, *Physical Review B*, 2000, **62**, 11556–11570.
